# A recombinant antibody against *Plasmodium vivax* UIS4 for distinguishing replicating from dormant liver stages

**DOI:** 10.1186/s12936-018-2519-7

**Published:** 2018-10-17

**Authors:** Carola Schafer, Nicholas Dambrauskas, Ryan W. Steel, Sara Carbonetti, Vorada Chuenchob, Erika L. Flannery, Vladimir Vigdorovich, Brian G. Oliver, Wanlapa Roobsoong, Steven P. Maher, Dennis Kyle, Jetsumon Sattabongkot, Stefan H. I. Kappe, Sebastian A. Mikolajczak, D. Noah Sather

**Affiliations:** 1 Center for Infectious Disease Research, Seattle, WA USA; 2 Mahidol Vivax Research Unit, Faculty of Tropical Medicine, Mahidol University, Bangkok, Thailand; 3 Center for Tropical and Emerging Global Diseases, University of Georgia, Athens, GA USA; 4 Present Address: Seattle Children’s Research Institute, Seattle, WA USA; 5 Present Address: Novartis Institute for Tropical Diseases, Emeryville, CA USA; 6 Department of Global Health, University of Washington, Seattle, USA; 7 Present Address: The Walter and Eliza Hall Institute of Medical Research, Parkville, Australia

**Keywords:** *Plasmodium vivax*, UIS4, Hypnozoite, Recombinant antibody

## Abstract

**Background:**

*Plasmodium vivax* is the most geographically widespread of the human malaria parasites, causing 50,000 to 100,000 deaths annually. *Plasmodium vivax* parasites have the unique feature of forming dormant liver stages (hypnozoites) that can reactivate weeks or months after a parasite-infected mosquito bite, leading to new symptomatic blood stage infections. Efforts to eliminate *P. vivax* malaria likely will need to target the persistent hypnozoites in the liver. Therefore, research on *P. vivax* liver stages necessitates a marker for clearly distinguishing between actively replicating parasites and dormant hypnozoites. Hypnozoites possess a densely fluorescent prominence in the parasitophorous vacuole membrane (PVM) when stained with antibodies against the PVM-resident protein Upregulated in Infectious Sporozoites 4 (PvUIS4), resulting in a key feature recognizable for quantification of hypnozoites. Thus, PvUIS4 staining, in combination with the characteristic small size of the parasite, is currently the only hypnozoite-specific morphological marker available.

**Results:**

Here, the generation and validation of a recombinant monoclonal antibody against PvUIS4 (α-rUIS4 mAb) is described. The variable heavy and light chain domains of an α-PvUIS4 hybridoma were cloned into murine IgG1 and IgK expression vectors. These expression plasmids were co-transfected into HEK293 cells and mature IgG was purified from culture supernatants. It is shown that the α-rUIS4 mAb binds to its target with high affinity. It reliably stains the schizont PVM and the hypnozoite-specific PVM prominence, enabling the visual differentiation of hypnozoites from replicating liver stages by immunofluorescence assays in different in vitro settings, as well as in liver sections from *P. vivax* infected liver-chimeric mice. The antibody functions reliably against all four parasite isolates tested and will be an important tool in the identification of the elusive hypnozoite.

**Conclusions:**

The α-rUIS4 mAb is a versatile tool for distinguishing replicating *P. vivax* liver stages from dormant hypnozoites, making it a valuable resource that can be deployed throughout laboratories worldwide.

## Background

Half of the worlds’ population is at risk of malaria infection. While *Plasmodium falciparum* is the predominant cause of malaria in Africa [1], *Plasmodium vivax* has the widest geographical distribution and is estimated to be responsible for nearly half the cases of malaria outside of sub-Saharan Africa, leading to 50,000–100,000 deaths annually [2].

Infection is initiated with the bite of an infected female *Anopheles* mosquito, which injects tens to hundreds of motile sporozoites into the skin [3]. The sporozoites traverse skin and endothelial cells to gain access to the blood circulation through which they are transported to the liver [4]. Once in the liver, sporozoites are sequestered in the sinusoids and enter the liver parenchyma where they infect hepatocytes which marks the beginning of the asymptomatic liver stage infection [5]. Approximately 7–9 days after sporozoite infection, tens of thousands of exo-erythrocytic merozoites are released from each infected hepatocyte and enter the bloodstream to infect human red blood cells. The following erythrocytic stage of infection, in which the number of parasites increases exponentially as well as ensures transmission to the mosquito vector, is responsible for all the clinical symptoms associated with malaria [6].

The pre-erythrocytic stage is a favourable target for intervention strategies, as preventing the release of exoerythrocytic merozoites from the liver would stop the disease before the onset of clinical symptoms and would prevent transmission. Also, the liver stages of *Plasmodium* do not develop drug resistance like it has been reported for the blood stages, likely due to a lower burden of liver parasites (10–10^2^) as compared to blood stage parasites (10^9^–10^13^) [7]. Importantly, it is at the liver stage where *P. vivax* differs greatly from *P. falciparum*. *Plasmodium vivax* forms dormant liver stages, termed hypnozoites, that create a reservoir of non-replicating, persistent parasites. These re-activate periodically and lead to new symptomatic blood stage infections, termed relapses, without new exposure to parasite-infected mosquitoes [8]. Remarkably, it has been reported that 80–90% of *P. vivax* infections are due to relapses and not to newly acquired infections [9]. Primaquine is the only drug that has been approved for preventing relapse of *P. vivax* infection. However, incompatibility with glucose-6-phosphate-dehydrogenase (G6PD) deficiency, treatment failures associated with decreased cytochrome P450-2D6 activity, and primaquine’s short half-life and long dosage regimens combine to diminish its usefulness in mass elimination campaigns [10, 11]. Thus, the potential for long-term *P. vivax* latency and lack of a safe, efficacious, single-dose drug effective against hypnozoites threatens the World Health Organization (WHO) goals of reducing malaria incidence and mortality rates by 90% and eliminating the disease from 35 endemic countries in the next 15 years [2].

The development of new research technologies, including in vitro infection of primary hepatocytes [12] and in vivo liver stage infections of liver-chimeric mice [13] has bolstered efforts to generate improved hypnozonticidal anti-malarials and liver stage-targeted vaccines. However, a critical point in both the in vitro and in vivo liver stage models of *P. vivax* is distinguishing between dormant hypnozoites and replicating liver stages. It was shown previously that hypnozoites can readily be distinguished from replicating liver stages by staining with an antibody against PvUIS4 (Upregulated in Infectious Sporozoites 4) [13]. UIS4 localizes to the parasitophorous vacuole membrane (PVM), a prominent feature of all *Plasmodium* liver stages which separates the parasite from the host cell cytoplasm. Although it is expressed in all liver stages, the staining pattern of *P. vivax* hypnozoites reveals a polarized, densely fluorescent prominence of the PVM which, in sections of infected chimeric mouse livers, has not been observed in developing liver stage parasites. Therefore, the UIS4-positive PVM prominence, together with the size of the parasite, enables the clear delineation between replicating liver stages and hypnozoites, a prerequisite for all research on *P. vivax* liver stages, and is currently the only known hypnozoite marker. It was recently reported that parasites cultured in vitro also showed a densely-fluorescent staining on a subpopulation of large liver stage parasites, but this staining pattern can easily be distinguished from a true prominence, as observed on hypnozoites [14].

Here, the generation and application of a recombinant monoclonal antibody against PvUIS4 (α-rUIS4 mAb) to help distinguish hypnozoites from developing liver stages is described. In general, recombinant monoclonal antibodies have various advantages over traditional hybridoma-derived monoclonal or polyclonal antibodies, like low batch-to-batch variation combined with high purity and affinity. Additionally, production of large quantities is more feasible and therefore reagents can be shared among groups interested in comparing and validating results. As described above, research on *P. vivax* relies solely on variable patient isolates. Therefore, to reliably compare experimental data, consistency within analytical reagents is of the utmost importance. As to date very little is known about hypnozoites, it is critical to standardize the tools used for the identification of this dormant parasite form.

As shown here, the α-rUIS4 mAb consistently detects PvUIS4 in varying different *P. vivax* patient isolates, and on both in vitro and in vivo samples. Because it robustly stains the hypnozoite-specific UIS4-positive prominence, it is a useful research tool and will be of significant interest and utility within the field.

## Methods

### Amplification of α-rUIS4 mAb from hybridoma

Hybridoma cells were produced by Genscript (Piscataway Township, NJ, USA) from Balb/c mice immunized with PvUIS4 peptide (amino acid sequence: VKHRKKARMEMDEPFTDLGEPIQIKKETNPFKMTSETLKPEVIVTTQRATGQMSRPSVIGEDVDGDTGENLGDSFFDKSFKPNVMGSPI). Cryopreserved hybridoma cells at 2 × 10^6^ cells/mL were placed in a 37 °C water bath until halfway thawed, then mixed with 10 mL of 37 °C RPMI (Corning, Corning, NY, USA) + 10% FBS (Gemini Bio-Products, West Sacramento, CA, USA). The cells were centrifuged at 300×*g* for 7 min, and resuspended in 350 μL RLT lysis buffer (Qiagen, Hilden, Germany) supplemented with 3.5 μL 2-mercaptoethanol. Cells were passed through a QIAshredder column to thoroughly disrupt the cells, and RNA was extracted using the AllPrep DNA/RNA Mini Kit (Qiagen, Hilden, Germany) according to the manufacturer’s protocol. 5′RACE-ready cDNA was generated using the SMARTer RACE 5′/3′ Kit (Takara-Clontech, Mountain View, CA, USA) according to the protocol, with the addition of gene-specific murine IgG and IgK primers (Mm IgG: GGGAAGTAGCCCTTGACCAGGC and Mm IgK: CCAGATGTTAACTGCTCACTGG).

Following cDNA synthesis, the reaction was diluted with 30 μL of Tris–EDTA from the SMARTer RACE kit. PCR was performed using 2 μL of the diluted cDNA as a template, 22.5 μL AccuPrime *Pfx* SuperMix (Thermo Fisher, Waltham, MA, USA), and 120 nM of forward (TCGTCGGCAGCGTCAGATGTGTATAAGAGACAGAAGCAGTGGTATCAACGC) and gene-specific IgG and IgK primers (Mm IgG CH1 R: GTCTCGTGGGCTCGGAGATGTGTATAAGAGACAGCAGGGGCCAGTGGATAGAC, Mm IgK CH1 R: GTCTCGTGGGCTCGGAGATGTGTATAAGAGACAGGGATACAGTTGGTGCAGC) plus water to a final volume of 25 μL. PCR cycling conditions were as follows: 98 °C–30 s; 5 cycles–98 °C–10 s, 50 °C–20 s, 68 °C–30 s; 5 cycles–98 °C–10 s, 53.5 °C–20 s, 68 °C–30 s; 30 cycles–98 °C–10 s, 57 °C–20 s, 68 °C–30 s followed by a 10-min, final extension at 68 °C. PCR amplicons were gel purified using FlashGel™ recovery gels (Lonza, Wakersville, MD, USA), cloned into a vector using the Zero Blunt TOPO PCR Cloning Kit (Thermo Fisher, Waltham, MA, USA), and subsequently transformed into One Shot TOP10 competent cells (Invitrogen, Carlsbad, CA, USA) according to the manufacturer’s instructions. These cells were grown overnight and plasmid DNA was isolated from single colonies using a QIAprep Spin Miniprep Kit (Qiagen, Hilden, Germany) followed by sequencing to confirm productive rearrangement of the variable region.

### Gibson assembly into expression vectors

Using the variable region sequence, unique primers corresponding to the IgHV and IgKV leaders were designed in order to incorporate overlapping vector sequence on the 5′ ends of the variable regions, in preparation for Gibson assembly (PvUIS4.IgG1 Gibson: ACAGGCGTGCACAGCGAGGTGCATCTGGTGGAG; PvUIS4.IgK Gibson: ACCGACGCTAGATGCGACATCCAGATGACACAG). Reverse primers incorporated overlapping vector sequence on the 3′ ends of the IgHV and IgKV constant regions. The constructs were amplified using Phusion Hot Start Flex 2X Master Mix (NEB, Ipswitch, MA, USA) with 120 nM of both the unique 3′ primer and the universal IgG1 or IgK 5′ primer. PCR cycling conditions were as follows: 98 °C–30 s; 30 cycles–98 °C–7 s, 64.5 °C–20 s, 72 °C–20 s followed by a 5-min, final extension at 72 °C. Amplicons were visualized and gel purified as described above. Expression vectors (similar to those used in [15]) encoding murine leader sequences (IgG: MEWSWVFLFFLSVTTGVHS, IgK: MGVPTQVLGLLLLWLTDARC) and the constant regions of either IgK (4IJ3_B, residues 107–214) or IgG1 (BAQ25543, residues 151–460) were linearized (using a unique site between the leader and constant-region coding sequences), and gel purified. Gibson assemblies were performed using 2X HiFi DNA Assembly Master Mix (NEB, Ipswitch, MA, USA), following the manufacturer’s instructions. Briefly, 2X HiFi was added to the purified variable region amplicon and the linearized vector, incubated at 50 °C for 1 h, then transformed into TOP10 cells. Single colonies were grown in LB culture overnight and the plasmid DNA was isolated by miniprep, then sequenced to verify the presence of a continuous open reading frame.

### Expression and purification of α-rUIS4 mAb

Plasmid DNA encoding the heavy and light chains of the recombinant antibody were co-transfected into HEK293 suspension cells for the expression of the mature recombinant antibody. For the production scale of 2 L, 900 μg of each plasmid encoding the heavy and light chains (1800 μg total) along with 1800 μL of PEI at 2 mg/mL (Polysciences Inc., Warrington, PA, USA) were added to 100 mL of HEK293 cells at a density of 20 × 10^6^ cells/mL. This culture was incubated for 3 h at 37 °C, 5% CO_2_ on a platform shaking at 135 rpm, then diluted to 1 × 10^6^ cells/mL for a final volume of 2 L and returned to incubate for 5 days. Cells were removed from harvested culture by centrifugation at 4000 rpm for 20 min at 4 °C. The clarified supernatant was adjusted to pH 6.0 with acetic acid and passed over Protein G Sepharose resin (GE Healthcare, Chicago, IL, USA) at 1 mL/min flow rate. Bound antibody was eluted with 0.1 M glycine, pH 2.7, and immediately neutralized with 1 M dibasic sodium phosphate. The purified antibody was immediately buffer exchanged into PBS, pH 7.4 with 0.02% sodium azide, and verified by size exclusion chromatography (SEC) on a Superdex 200 10/300 column (GE Healthcare, Chicago, IL, USA). The antibody was also visualized by SDS-PAGE on a NuPage 4–12% Bis–Tris gel (Invitrogen, Carlsbad, CA, USA). 2 μg of purified antibody was mixed with either NuPAGE LDS Sample Buffer (Invitrogen, Carlsbad, CA, USA) or with LDS Sample Buffer + 2.5% 2-mercaptoethanol, non-reduced and reduced conditions respectively, and incubated at 100 °C for 5 min. PageRuler Plus Prestained Protein Ladder (Invitrogen, Carlsbad, CA, USA) was run next to the antibody, for size comparison. Following SDS-Page, the gel was stained using SimplyBlue SafeStain (Invitrogen, Carlsbad, CA, USA), de-stained with distilled water, and imaged using a FluorChem E system (ProteinSimple, San Jose, CA, USA).

### Antigen binding by α-rUIS4 mAb

Synthetic peptide corresponding to PvUIS4 (PlasmoDB:PVX_001715 amino acids 78–166) was produced by Genscript (Piscataway, NJ). Monoclonal antibody binding was detected using an Octet QK^e^ instrument (FortéBio, Inc, Menlo Park, CA, USA). Briefly, the antibody was diluted in 10× Kinetics Buffer (10× KB: 1× PBS supplemented with 0.02% Tween-20, 0.1% bovine serum albumin, and 0.05% sodium azide) to a final concentration of 10 µg/mL and coupled to Dip and Read™ Anti-Mouse IgG Fc Capture (AMC) Biosensors (FortéBio, Inc, Menlo Park, CA, USA). Biosensors were then incubated in 10× KB solutions containing the PvUIS4 peptide (concentration ranging from 33 to 1 nM) for 400 s (to observe association), and then transferred to wells containing 10× KB for 100 s (to observe dissociation). Data were processed using the ForteBio Data Analysis Software (version 7.0.1.2) with a simple 1:1 binding model to yield estimated on- and off-rates, as well as the derived K_D_ estimates.

### Hepatocyte culture

#### Primary human hepatocytes (PHH) in 384-well plates

The day prior to hepatocyte seeding wells were coated with 40 μL of 15 μg/μL rat tail collagen I (BD) in filter-sterilized 0.02 N acetic acid, and then left overnight at 37 °C. Immediately prior to seeding, the collagen solution was washed out thrice with sterile PBS and then filled with 20 μL In Vitro GRO^®^CP plate media (Bioreclamation IVT, Westbury, NY, USA) supplemented with 1× Pen-Strep-Neo solution (Gibco, Thermo Fisher, Waltham, MA, USA) and 20 μM gentamicin (Gibco, Thermo Fisher, Waltham, MA, USA). Vials of cryopreserved PHH (Bioreclamation IVT, Westbury, NY, USA) were thawed by immersion in a 37 °C water bath for 2 min, sterilized by 70% ethanol in a sterile field, and contents added directly to 4 mL plate media. Live and dead cells were quantified by trypan blue exclusion on a Neubauer improved haemocytometer, the hepatocyte density was set to 1000 live cells/μL, and 18 μL cell suspension was added to each well. Media was exchanged thrice weekly with In Vitro GRO^®^CP plate media.

#### Primary human hepatocytes in 8-well chamber slides

The day before plating, permanox 8-chamber slides (LabTek, Thermo Fisher) were coated with 100 µg/mL bovine type-I collagen (Advanced BioMatrix, Carlsbad, CA, USA) in nanopore water supplemented with 130 µg/mL *N*-Cyclohexyl-*N*′-(2-morpholinoethyl)carbodiimide methyl-*p*-toluenesulfonate (CMC; Sigma Aldrich, St. Louis, MO, USA #C106402). Slides were incubated with the coating solution at 37 °C for 2 h, the morning after which the solution was aspirated, washed once with sterile PBS, and dried at 37 °C overnight. The day of plating, each well of the chamber slide was filled with 300 µL of InVitroGRO™ CP media (Bioreclamation IVT) without antibiotics. Vials of primary human hepatocytes (Bioreclamation IVT) were then thawed, live cells enumerated, and 200 µL of cell suspension containing 1.4 × 10^5^ live cells plated in each well according to the manufacturer’s instructions. Cultures were allowed to adhere at 37 °C for 4 h before plating media containing dead and unattached cells was removed and replaced with complete InVitroGRO™ CP media supplemented with Torpedo™ Antibiotic Mix (Bioreclamation IVT) and 10 µg/mL gentamycin (Gibco, Thermo Fisher).

#### HCO_4_ cells

HCO_4_ cells were maintained in MEM + F12 media (Gibco) containing 10% heat inactivated fetal bovine serum and penicillin/streptomycin (100 units/100 μg/mL; Gibco) in T75 cm^2^ flask. Cells were subcultured by trypsinization with 0.125% Trypsin/EDTA once the confluency has reached 80%.

### Infection and staining of in vitro hepatocyte cultures

All sporozoites were obtained from mosquitoes that had been fed on *P. vivax* infected blood obtained from patients seeking treatment for *P. vivax* malaria at various malaria clinics along the Thai–Myanmar border. After 14–16 days of mosquito-stage development, mosquito salivary glands were dissected and sporozoites were collected into 100 μL cold Schneider’s insect media (Sigma Aldrich) or RPMI (Gibco, Thermo Fisher). Sporozoite density was counted on a Neubauer improved haemocytometer.

#### Primary human hepatocytes in 384-well plates

Sporozoite density was adjusted to 250 sporozoites/μL in complete InVitroGRP™ CP media, and 5000 sporozoites were added to wells. Freshly inoculated cultures were then spun at 200 RCF for 5 min and media exchanged the next day and thrice weekly thereafter. At the desired time point, cells were fixed using 4% paraformaldehyde followed by two PBS washes. The α-rUIS4 mAb was diluted 1:25,000 in dilution buffer (1% w/v Bovine Serum Albumin and 0.3% v/v Triton X-100 in PBS) and cells were stained over night at 4 °C. Primary antibody stained cultures were washed thrice with PBS and secondary stained with 2 μg/mL Alexa Fluor 488-lableled goat anti-mouse IgG (Thermo Fisher) and 10 μg/mL Hoechst 33342 (Sigma Aldrich) to visualize nuclei.

#### Primary human hepatocytes in 8-well chamber slides

Two days after plating, sporozoites were prepared to a concentration of 700 sporozoites/µL in complete InVitroGRP™ CP media supplemented with 20% FBS, and 1.4 × 10^5^ sporozoites added to each well before spinoculation at 500×*g* for 3 min. Inoculated cultures were incubated at 37 °C before media was changed 4 h later, and daily thereafter with complete InVitroGRP™ CP media. On day 6 post infection, cells were washed twice in PBS and subsequently fixed in 10% formalin for 30 min at room temperature. After fixing, the cells were washed three times in PBS before blocking and permeabilization in PBS containing 2% BSA and 0.2% Triton X-100 (blocking solution) for 1 h at room temperature. The α-rUIS4 mAb was diluted 1:10,000 in blocking solution, while the parental, hybridoma-derived monoclonal antibody was diluted 1:500 in blocking solution and incubated with samples overnight at 4 °C. The next day, slides were washed three times in PBS before addition of 2 μg/mL Alexa Fluor 488—labeled goat anti-mouse or goat anti-rabbit IgG (Thermo Fisher) in blocking buffer for 2 h at room temperature, then stained with 1 µg/mL DAPI to visualize nuclei before mounting using Prolong Gold Antifade reagent to preserve fluorescence.

#### HCO_4_ cells

HCO_4_ cells were seeded at 25,000 cells/well 24 h prior to sporozoite infection. Sporozoites were prepared to a concentration of 2000 sporozoites/µL and 25 μL of sporozoite suspension (50,000 sporozoites) were added to each well before spinoculation at 2000 rpm for 5 min. The culture plate was maintained at 37 °C with daily media changes for 7 days. On day 8, cells were fixed in 4% PFA for 1 h at room temperature, then washed three times with 1× PBS and permeabilized in PBS containing 1% Triton X-100 for 30 min at room temperature. Cells were washed three times in PBS before blocking in 3% BSA in PBS for 1 h at room temperature. Subsequently, parasites were stained with rUIS4 mAb directly conjugated to Alexa Fluor 488, diluted to 1:6500, and 1 μg/mL DAPI at 4 °C overnight.

### In vivo infection and liver immunofluorescence assays

FRG huHep mice were intravenously injected with one million *P. vivax* sporozoites. Livers were harvested on day 8 post-infection and fixed in 10% formalin for 24 h before they were sliced to 50 μm sections with a vibratome. Liver sections were permeabilized in 10% H_2_O_2_ and 0.25% Triton X-100 in 1× TBS for 30 min at room temperature with agitation, then washed in 1× TBS and blocked in 5% milk in 1× TBS for 1 h at room temperature. The primary antibodies, α-rUIS4 mAb and HSP60 mAb, were diluted to 1:500 in 5% milk in 1× TBS and liver sections were incubated over night at 4 °C with agitation. After primary antibody staining, liver sections were washed five times in 1× TBS for 5 min per wash before addition of 2 μg/mL Alexa Fluor 488-labeled goat anti-mouse IgG (Thermo Fisher) and Alexa Fluor 594-labeled goat anti-rabbit IgG (Thermo Fisher) for 2 h at room temperature. Liver sections were again washed five times for 5 min per wash before they were transferred to 1 mL 0.06% KMnO_4_ in H_2_O. After another wash in 1× TBS, cells were stained in 2 μg/mL DAPI for 5 min at room temperature. After washing in 1× TBS, sections were mounted using Prolong Gold Antifade reagent to preserve fluorescence.

### Microscopy

High-magnification (60×, 100×) images of LS parasites in primary human hepatocytes were acquired on a Deltavision Elite deconvolution microscope (GE Healthcare Lifesciences) and processed using softWoRx^®^ deconvolution software (GE Healthcare Lifesciences).

## Results

### Antibody production

**Fig. 1:**
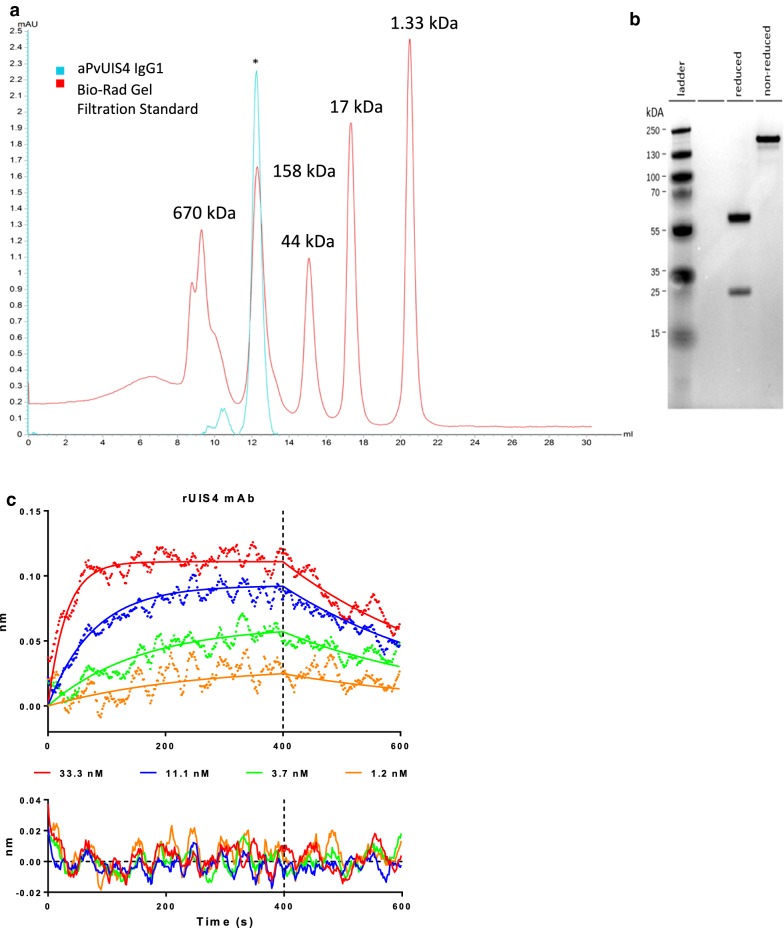
Quality control of α-rUIS4 mAb. **a** SEC (Superdex Increase 200 10/300 GL) trace of α-rUIS4 IgG1 (blue) and Bio-Rad Gel Filtration Standards (Red). *Y*-axis, absorbance (280 nm), *X*-axis, elution volume (mL). The asterisk indicates the antibody peak. **b** 4–12% Bis–Tris SDS-PAGE analysis of α-rUIS4 IgG1 under reduced (left) or non-reduced (right) conditions. **c** Biolayer interferometry analysis of rUIS4 mAb binding to the immunogen corresponding to the cytoplasmic sequence of *P. vivax* UIS4 in the concentration range of 33–1 nM. The top panel shows biphasic fits of the binding curves while the bottom panel shows the residuals of the fits

To generate a reagent for the reliable detection of hypnozoites, total RNA was extracted from an anti-PvUIS4 hybridoma that was previously described [13]. The coding regions of the heavy and light chains were rescued by 5′RACE using primers with specificity to the constant regions of murine IgG and IgK. Amplification of the heavy and light chains of the hybridoma generated a heavy chain amplicon of approximately 550 bp and a light chain of roughly 500 bp. Both amplicons were purified, cloned into pCR TOPO Zero Blunt, and sequenced. The productively rearranged heavy and light chains were cloned into IgG and IgK expression vectors by Gibson assembly. Sequence-validated expression vectors were transfected into HEK293 cells for expression and purification. Purified α-rUIS4 mAb was quantitatively analysed by analytical size exclusion chromatography (SEC) (Fig. 1a) and reducing/non-reducing SDS-PAGE assay (Fig. 1b) which indicated that the recombinant antibody expressed as a mature IgG is approximately 150 kDa in size. Additionally, the affinity of the α-rUIS4 mAb to the cognate rUIS4 immunogen derived from the cytoplasmic sequence of *P. vivax* UIS4 was tested by Octet BioLayer Interferometry (BLI) (Fig. 1c) and determined to be 3.6 ± 0.3 nM.

### α-rUIS4 mAb staining of in vitro samples

**Fig. 2:**
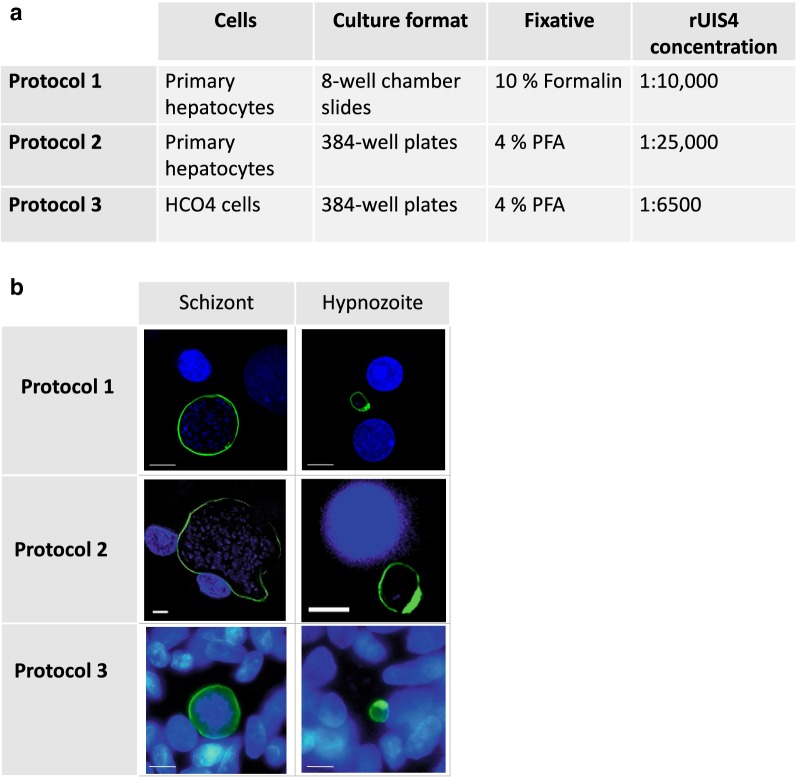
α-rUIS4 mAb staining in vitro. The α-rUIS4 mAb was used to stain liver stage schizonts and hypnozoites in three individual in vitro immunofluorescence assays (IFAs) on *P. vivax* infected hepatocyte cultures. Three different patient isolates were used for the infections. The differences between the culturing and staining protocols are depicted in **a**. **b** Representative images of liver stage schizonts and hypnozoites stained with the three different protocols. The UIS4-positive prominence is clearly visible on all hypnozoites, but not on liver stage schizonts, thereby allowing the differentiation between dormant and replicating parasite forms

Next, it was investigated whether the α-rUIS4 mAb can detect *P. vivax* liver stage parasites reproducibly across diverse parasite isolates and under different culture and staining conditions used in the *P. vivax* research community. To do so, three different in vitro protocols using three parasite isolates were compared and it was found that the α-rUIS4 mAb functions reproducibly throughout all of them (Fig. 2a, b).

The differences between the protocols are shown in Fig. 2a. Briefly, primary hepatocytes were cultured in 8-well chamber slides or 384-well plates and infected with different isolates of *P. vivax* sporozoites. HCO_4_ cells were cultured in 384-well plates and infected with sporozoites of a third *P. vivax* patient isolate. On day 6 post infection, the cells were fixed in 10% formalin or 4% paraformaldehyde, and permeabilized. The α-rUIS4 mAb was diluted 1:10,000, 1:25,000 or 1:6500 and cells were incubated over night at 4 °C. The α-rUIS4 mAb staining reveals a circumferential pattern on large forms, whereas the additional protein-dense prominence is stained on small forms, which allows the identification of these forms as hypnozoites. All three protocols showed similar results, as shown in Fig. 2b.

**Fig. 3:**
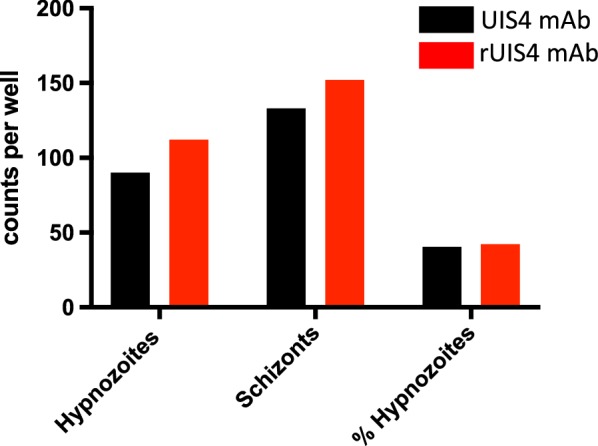
Comparison of liver stage schizont and hypnozoite counts using α-rUIS4 mAb or α-UIS4 hybridoma-derived mAb. One 8-well chamber slide of primary hepatocyte cultures was infected with *P. vivax* sporozoites. On day 8 post infection, cells were fixed and stained using Protocol 1, as described in Fig. 1. Cells in one well were stained with the α-rUIS4 mAb and another well on the same slide was stained with the parental hybridoma-derived monoclonal α-UIS4 antibody. Hypnozoite and liver stage schizont counts, as well as the hypnozoite to schizont ratios, were similar, indicating that there is no difference in staining efficiency between α-rUIS4 mAb and α-UIS4 hybridoma-derived mAb

To test whether α-rUIS4 mAb staining is comparable to the parental hybridoma-derived monoclonal α-UIS4 antibody (α-UIS4 mAb) [13], two wells of a *P. vivax* infected hepatocyte culture of an 8-well chamber slide were stained either with α-rUIS4 mAb or α-UIS4 mAb using the protocol described above. Schizonts and hypnozoites were counted and the percentage of hypnozoites as well as the hypnozoite:schizont ratios were calculated. Similar absolute counts as well as hypnozoite:schizont ratios were obtained for both antibodies (Fig. 3), indicating that the α-rUIS4 mAb stains schizonts and hypnozoites as efficiently as the parental α-UIS4 mAb. Additionally, recent publications using the parental monoclonal antibody [14] or the recombinant antibody [16] show that the two antibodies perform similarly.

### PvUIS4 localization in vivo in *Plasmodium vivax* infected livers

Liver-chimeric mice poise the unique opportunity to study *P. vivax* liver stages in vivo. Here, the FRG huHep mouse model was utilized, which was previously shown to support *P. vivax* liver stage development, hypnozoite formation and relapse [13], to test whether the α-rUIS4 mAb stains *P. vivax* parasites in formalin-fixed liver sections.

**Fig. 4:**
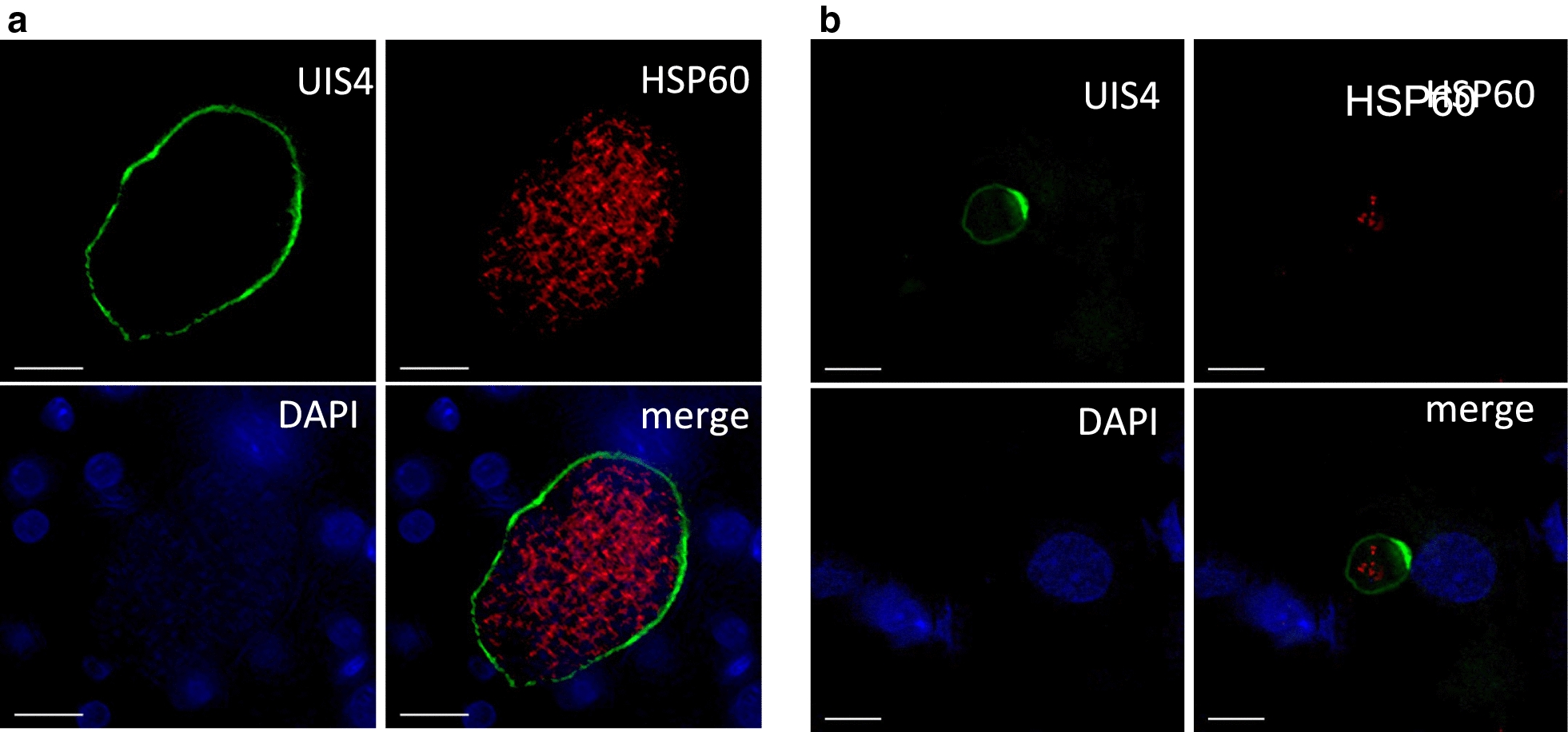
α-rUIS4 mAb staining in vivo. Liver-chimeric FRGN huHep mice were infected intravenously with one million *P. vivax* sporozoites. On day 8 post infection, livers were harvested, fixed and stained with α-rUIS4 mAb and the parasite mitochondrial marker anti-HSP60 as described above. DNA was stained with DAPI. **a** Representative image of a schizont in which α-rUIS4 mAb continuously stains the PVM. **b** Representative image of a hypnozoite in which the UIS4-positive prominence is clearly visible. Scale bars = 10 μm

FRG huHep mice were infected intravenously with one million *P. vivax* sporozoites. At day 8 post infection, when liver stage development is nearly complete, livers were harvested, fixed in 10% Formalin for 24 h, sliced to 50 μm sections with a vibratome and subsequently stained with the α-rUIS4 mAb. The liver sections were counter-stained with the parasite mitochondrial marker HSP60. Figure 4a shows a representative image of a mature schizont, where the α-rUIS4 mAb shows a consistent circumferential staining pattern, indicative of PVM localization. In contrast, the UIS4-positive prominence is clearly visible in the hypnozoite, as shown in Fig. 4b, indicating that the α-rUIS4 mAb is fully functional on formalin-fixed liver sections from liver-chimeric mice. This staining pattern is consistently seen on day 8 liver sections.

## Discussion

Researchers have been using antibodies as analytical reagents to study proteins of interest for decades [17]. Originally, polyclonal antibodies (pAbs) were used, which are produced by immunizing an animal with a specific target and using the resulting serum as a source of antibodies [18]. However, the antibodies in a polyclonal serum are widely variable and contain a diverse mix of target and off-target antibodies. Thus, in most cases only a small fraction of pAbs in any given analytical serum bind to their intended target. Moreover, immunizing an animal never produces the exact same mix of antibodies, which can lead to large batch-to-batch variation and the need for continual re-optimization within assays. A significant improvement was made by the introduction of monoclonal antibodies, which were originally made by fusing an antibody-producing B cell of an immunized animal with a cancer cell, thereby producing a hybridoma. The resulting hybridoma cell line, if properly managed, can be used to generate a single antibody species with high reproducibility. However, in 2008 a study revealed that fewer than half of around 6000 commercially available antibodies recognized only their specific target [19]. Also, hybridoma cells are only a finite source for antibodies, and must be carefully propagated and stored for maximal longevity. Therefore, in 2015 Andrew Bradbury, Andreas Plueckthun and 110 co-signatories called for the use of recombinant antibodies, which are defined by the sequence that encodes them and produced by expressing this sequence from plasmid DNA in cell lines [20]. In this way, it can be guaranteed that only a single recombinant antibody species is being produced and this method provides researchers with an infinite resource, as a specific antibody can be produced as often as needed and the sequence is preserved electronically if research materials are lost or destroyed.

For research on *P. vivax*, the use of recombinant antibodies is especially important. As *P. vivax* cannot be cultured in the laboratory, all research relies solely on patient samples, introducing significant variability into experimental systems. Therefore, to ensure robust comparisons in between experiments, the reagent has to be highly defined and proven to work across multiple field isolates. Additionally, validation of antibody-based findings by epitope tagging the target protein is currently not possible since transgenic parasites cannot be produced, due to the lack of a long-term in vitro blood stage culture system for *P. vivax*. This underscores the necessity for reliable and validated monoclonal antibodies in *P. vivax* research. Recombinant antibodies are of significant importance in this aspect, as they are highly specific, there is very little batch-to-batch variation (which can be quantitatively measured) and they bind their target with high affinity. For these reasons, recombinant α-PvUIS4 mAb was produced, which will be of great benefit for the *P. vivax* research community. It is shown here that it binds its target UIS4 with nanomolar affinity. Hypnozoite-specific staining of the UIS4-positive prominence, in combination with the size of the parasite, enables the clear differentiation between hypnozoites and replicating liver stages which is of paramount importance for any research conducted on *P. vivax* liver stages, as was recently shown by Roth et al. [16]. The observation of identical staining patterns by α-rUIS4 mAb within all four different *P. vivax* clinical isolates that were tested demonstrates that it is highly reproducible and allows comparisons in between experiments and laboratories, regardless of the source of the clinical isolate.

## Conclusion

The recombinant monoclonal antibody against PvUIS4 described here is a highly versatile tool for the study of *P. vivax* liver stages. It stains replicating liver stages and dormant hypnozoites, which allows reliable discrimination between the two forms due to the hypnozoite-specific UIS4-positive PVM prominence. Thus, the α-rUIS4 mAb will be of significant utility to the *P. vivax* liver stage research community.
